# A novel method for detection of camellia oil adulteration based on time-resolved emission fluorescence

**DOI:** 10.1038/s41598-018-32223-6

**Published:** 2018-09-13

**Authors:** Hui Chen, Bin Chen, Daoli Lu

**Affiliations:** School of Food and Biological Engineering, Jiangsu University, Zhenjiang, 212013 China

## Abstract

In this study, time-resolved emission fluorescence (TRES) combined with chemometrics was developed and employed for adulteration analysis of camellia oil. TRES was first decomposed by parallel factors analysis (PARAFAC). Next, an artificial neural network (ANN) model was built for the adulteration analysis. A linear range of 5–50%, a limit of detection (LOD) of 3% and root mean square error of prediction (*RMSEP*) values lower than 3% were achieved. Compared with the steady-state measurement, easy access to the information from fluorophores of low concentration was shown to be an intrinsic advantage of the time-resolved measurement; this advantageous characteristic was helpful for optimizing adulteration analysis. It was demonstrated that TRES combined with chemometrics was a simple, rapid and non-intrusive method for adulteration analysis of vegetable oil.

## Introduction

Camellia oil (CO) is recognized and consumed widely because of its highly nutritional value, health benefits and particular taste in subtropical Asia (China, India, Sri Lanka, Indonesia and Japan). It is widely considered as “Eastern olive oil” because its major fatty acids resemble those of olive oil^1^. CO contains squalene and sterols, which have been characterized as health promoting agents^2–5^. In addition, being rich in antioxidants (such as vitamins and polyphenols) makes CO a good additive to extend the shelf life of other oils^3,6^. All these factors have raised CO to a popular status; as a result, adulteration of CO has been conducted for economical purposes. The common adulterants are peanut oil (PO) and sunflower oil (SO) because of their low price. Both the interest of consumers and safety concerns deserve increasing attraction regarding CO adulteration.

The basic approach in adulteration analysis is to define one or more markers to check the purity of the specific oil^7^. Generally, these markers are chemical compounds that ideally should be absent in the original oil and present in the adulterated one^7^. However, methods focusing on specific compounds require either complex pretreatment or significant time consumption, both of which limit the application of such methods in rapid detection.

Fluorescence spectroscopy combined with chemometrics is a promising method for adulteration analysis and is characterized as sensitive, rapid and non-intrusive^8,9^. However, signals from fluorophores of low concentration are generally weak and can be easily masked by strong signals from other chemicals. This problem results in a performance degradation of the calibration model for adulteration analysis. Solutions to this problem, such as derivative and synchronous fluorescence, have been developed and employed. However, these current solutions are considered insufficient, given that they are limited to single fluorescence parameter, e.g., excitation or emission^10,11^. Additionally, steady-state measurement, which is a result of time average, is another reason accounting for this problem^12^.

As we know, fluorescence can be described by many parameters, such as excitation, emission and lifetime^13,14^. Analysis of fluorescence from a second parameter would add additional information to the spectrum. The additional information could improve the performance of the calibration model for adulteration analysis. Hence, time-resolved emission fluorescence (TRES), which is obtained by simultaneously measuring emission and decay of fluorescence, is considered as an effective method for adulteration analysis of vegetable oil. Additionally, many studies indicate that the analysis of TRES, which is based on time-resolved measurement, provides unique information compared with steady-state measurement^15–18^.

In this study, the application of TRES combined with chemometrics was performed for adulteration analysis of CO. Further investigation on fluorescence decay was performed to show the intrinsic advantage of TRES in adulteration analysis. The object of this study was to develop a simple, rapid and non-intrusive method for adulteration analysis while deepening our understanding of the time-resolved technique in food and other detection fields.

## Results and Discussion

### Investigation of the advantage of time-resolved measurement for adulteration analysis

Fluorescence measurements can be broadly classified into two types of measurements: steady-state and time-resolved^12^. Easy access to the information from fluorophores of low concentration was considered as an intrinsic advantage of time-resolved measurement and was helpful for optimizing adulteration analysis, as proved by the comparison between these two types of measurements.

**Figure 1:**
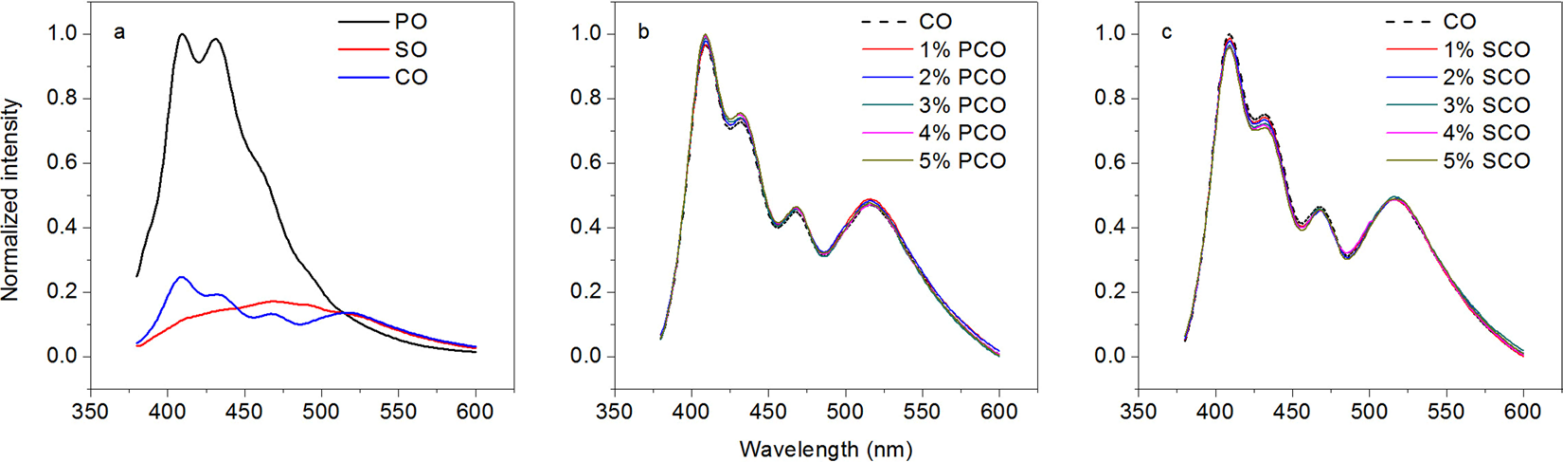
Equivalent emission spectra of pure oils (PO, SO and CO), (**a**) and adulterated oils (PCO and SCO, 1–5% level of adulteration, (**b**,**c**), respectively).

The equivalent steady-state emission spectra of pure oils (PO, SO and CO) and adulterated oils (PCO and SCO, 1–5% level of adulteration) are shown in Fig. 1. Significant differences in the fluorescence profile are observed among the equivalent steady-state emission spectra of pure oils (Fig. 1a). However, in comparison with CO, hardly any difference in the fluorescence profile could be observed in the equivalent steady-state emission spectra of PCO and SCO (Fig. 1b,c, respectively). This result can be easily understood by considering that steady-state fluorescence profile is usually dominated by fluorophores of high concentration, whereas signals from fluorophores of low concentration are easily masked. This masking was considered as a problem in adulteration analysis, especially when the adulteration amount is low and the fluorescence signals from adulterants (PO and SO) are masked. This problem is common in steady-state measurement as a result of the time averaging effect^12^. In particular, steady emission is treated as a homogenous process, leading to the resulting spectrum being a result of taking the time average. However, steady emission is an inhomogeneous process, and efficient extraction of signals from adulterants (PO and SO) is useful for optimizing adulteration analysis, especially in low adulteration amount.

**Figure 2:**
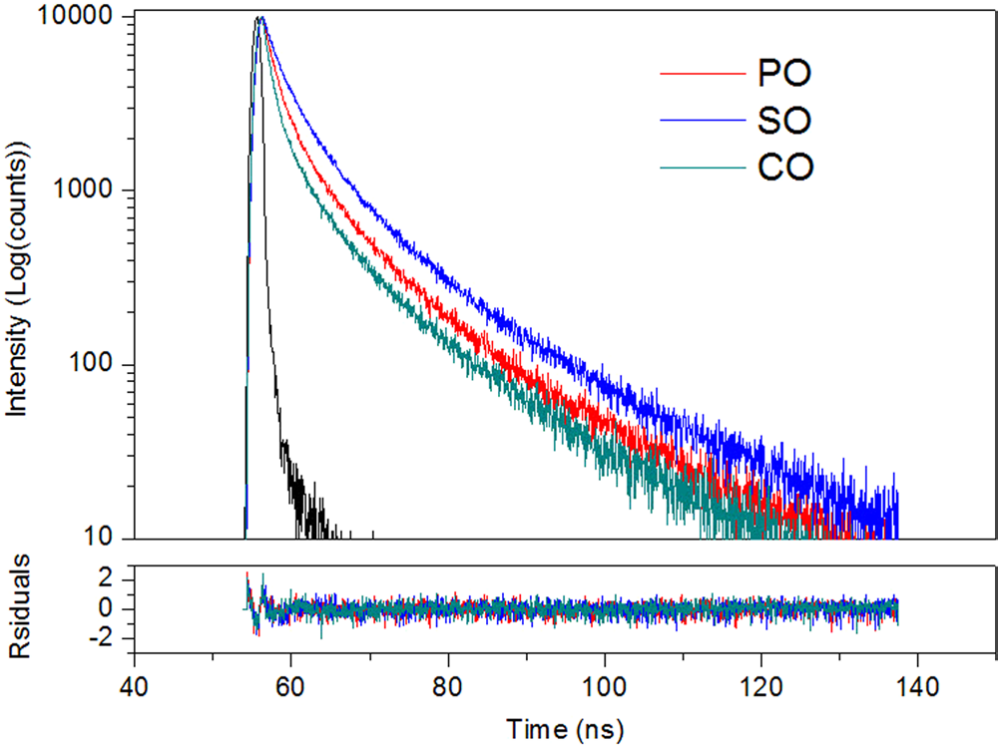
Fluorescence decay spectra of PO, SO and CO at 440 nm emission wavelength.

For the time-resolved measurement, significant differences are observed in the fluorescence decays of pure oils at 440 nm emission, as shown in Fig. 2. Note that the fluorescence decay time of PO and SO is longer than that of CO, despite their fluorescence intensities being almost the same at the initiation of decay. This phenomenon indicated that steady emission is an inhomogeneous process. For further extraction of information from the fluorescence decay, reconvolutions of the fluorescence decays were performed for two, three, and four components. The best fits for PO, SO and CO were found to be with three components. All of the calculated data are given in Table 1. Table 1 indicates that lifetimes of the longest-lifetime components (τ_3_) in PO and SO are longer than that of CO, whereas the longest-lifetime components are the lowest in concentration (f_3_) in pure oils. However, the signals from the longest-lifetime components were successfully extracted by the measurement of fluorescence decay via the intrinsic lifetime differences of fluorophores. In principle, fluorescence decay is associated with fluorescence lifetime, which is mainly dependent on the species of the fluorophore and is independent of the fluorophore concentration^18^. Hence, despite the adulteration amount being low, measurement of fluorescence decay is considered to be effective in obtaining signals from adulterants (PO and SO) for optimizing adulteration analysis.

**Table 1:** Fluorescence decay parameters of PO, SO and CO at 440 nm emission wavelength.

	τ_1_ (ns)	f_1_ (%)	τ_2_ (ns)	f_2_ (%)	τ_3_ (ns)	f_3_ (%)	χ^2^
PO	1.57 ± 0.01	38.79	6.14 ± 0.02	42.18	18.66 ± 0.01	19.04	0.9924
SO	2.25 ± 0.01	31.28	6.85 ± 0.01	46.37	19.85 ± 0.02	21.81	1.0137
CO	1.09 ± 0.02	41.65	5.05 ± 0.01	37.32	16.43 ± 0.01	21.02	1.0077

Component lifetime (τ), fluorescence contribution rate of component at certain wavelength (f), goodness of fit (χ^2^).

### TRES of pure oils and adulterated oils

**Figure 3:**
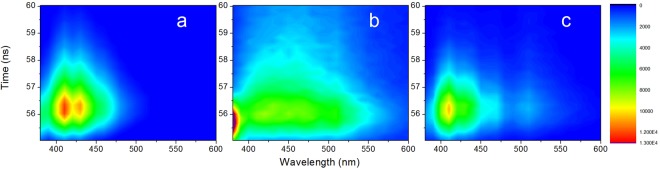
TRES spectra of PO (**a**), SO (**b**) and CO (**c**).

**Figure 4:**
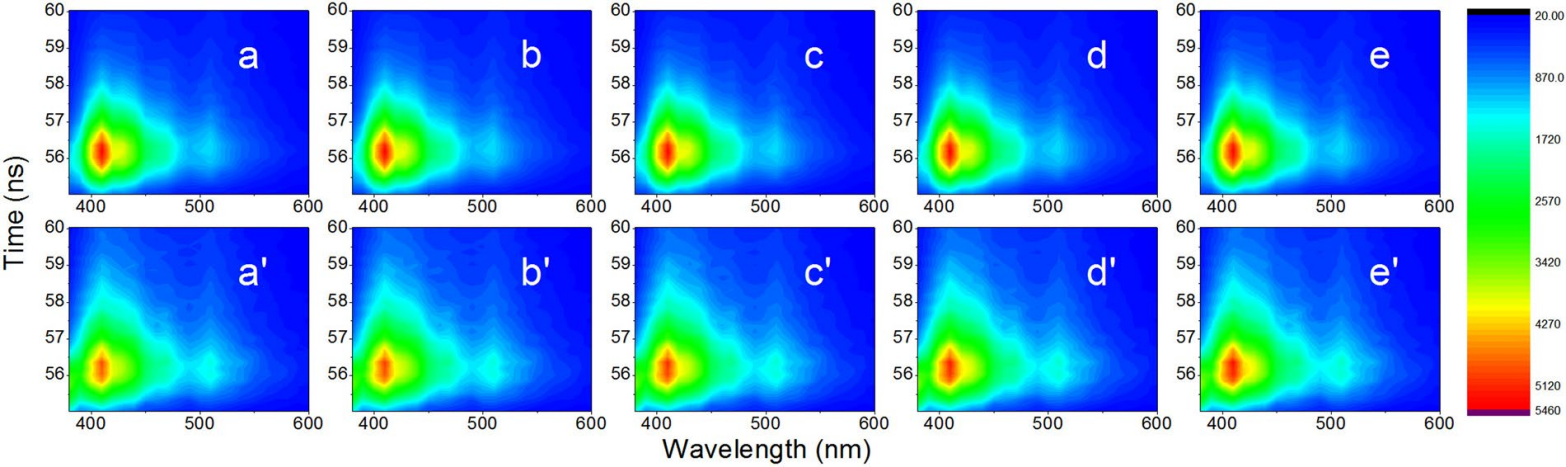
TRES spectra of PCO (**a**–**e**) and SCO (a’–e’), 1–5% level of adulteration.

TRES spectra of PO, SO and CO are shown in Fig. 3, and those of PCO and SCO (1–5% level of adulteration) are shown in Fig. 4. Longer fluorescence decays are observed in the TRES spectra of PO and SO compared with those of CO from the time dimension. In addition, similar long fluorescence decays are also obvious in the TRES spectra of PCO and SCO, despite the low levels of adulteration (1–5%). As mentioned above, measurement of fluorescence decay is considered to be effective in obtaining signals for optimizing adulteration analysis. Hence, despite the adulteration amount being very low and the resulting fluorescence intensities of TRES spectra between pure oil (CO) and adulterated oils (PCO and SCO) and within groups (PCO and SCO, respectively) observed from emission dimension being similar with each other (More clearly shown in Fig. 1b,c), feature signals from the time dimension were successfully extracted for optimizing adulteration analysis. Thus, TRES, which is a full-wave band measurement of fluorescence decay, was considered to be able to supply more feature signals for optimizing adulteration analysis.

### PARAFAC results

The results of core consistency diagnosis are shown in Fig. S1 (Supplementary data). The appropriate numbers of factor m are both four for PCO and SCO. TRES arrays were successfully decomposed by parallel factors analysis (PARAFAC), and the sample scores were set as the input of artificial neural network (ANN).

### Adulteration analysis

**Figure 5:**
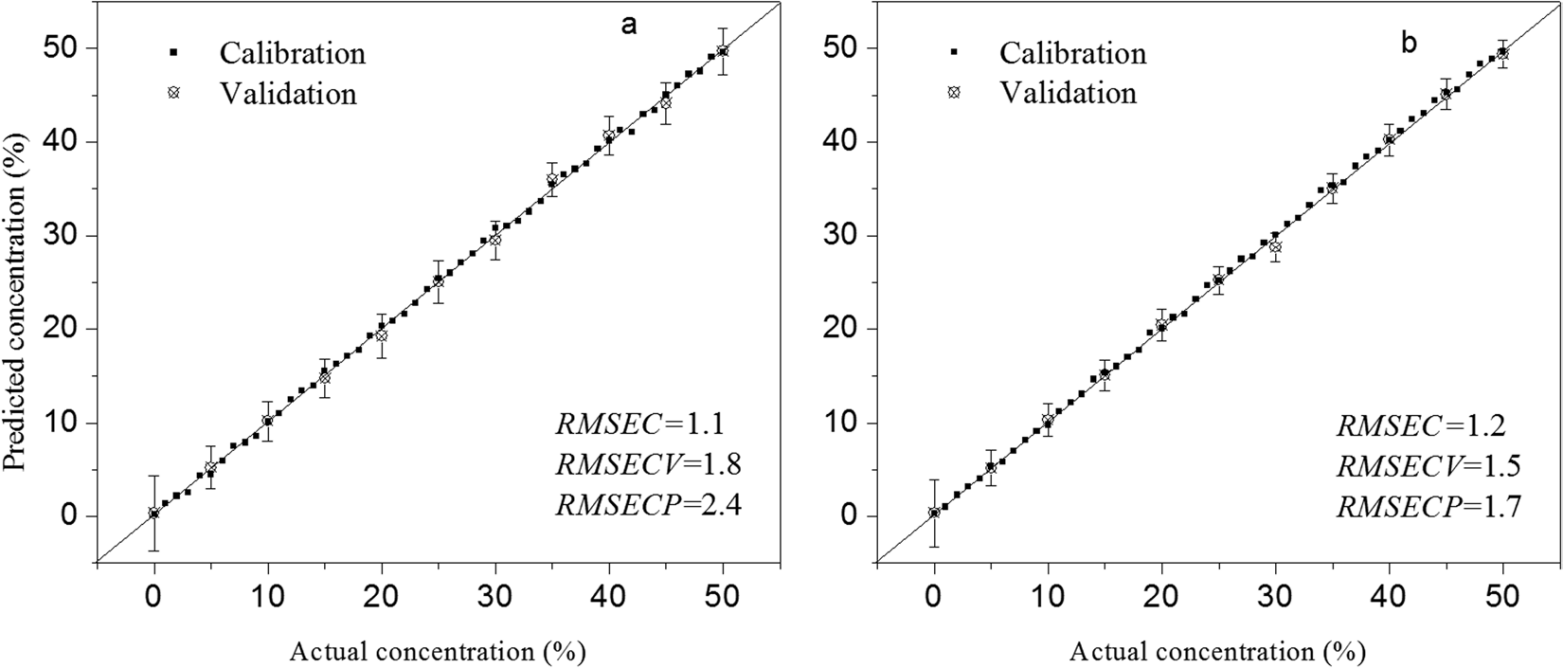
Predicted versus actual concentration of PCO (**a**) and SCO (**b**).

ANN models were built for adulteration analysis^19^. The results are shown in Fig. 5, Table S2 and Equation S1 (Supplementary data). The predicted and observed values display high linearity, as shown in Fig. 5. The linear range is 5–50% and the limit of detection (LOD) is 3% for both PCO and SCO. The values of ${R}_{cv}^{2}$ and ${R}_{p}^{2}$ are at least 0.96 and 0.97 for PCO and SCO, respectively. The values of *RMSECV* and *RMSECP* are lower than 3% and 2% for PCO and SCO, respectively. These results demonstrated that TRES combined with chemometrics was an effective method for adulteration analysis at low levels of adulteration concentration.

## Theoretical Considerations

### Trilinear property of TRES

The trilinear property of TRES should be proved first because PARAFAC is only suitable for the decomposition of three-way data, in which the corresponding instrumental response must obey Beer’s law. This task can be accomplished by considering the following equation. Similar to steady-state fluorescence, fluorescence decay at the detection time d*t* is the linear superposition of each fluorophore’s fluorescence; thus Eqn. (1) can be derived as follows:

1 $$S(\lambda ,{\lambda }_{0})={\sum }_{k}\int \frac{L(t){\rm{d}}t{\lambda }_{0}}{hc}{N}_{k}{\sigma }_{k}{\rm{\Delta }}R\xi \eta (\lambda ){\int }_{0}^{{\rm{\Delta }}R}{e}^{-({\alpha }_{{\lambda }_{0}}+{\alpha }_{\lambda })}d{r}$$

where $S(\lambda ,{\lambda }_{0})$ denotes the number of fluorescence photon detected at the detection time d*t* (s) at *λ*_ex_ =* λ*_0_ and *λ*_em_ =* λ*. k denotes the fluorophore fluorescing at *λ*_em_ =* λ*. *L*(*t*) denotes the laser energy (J). $\frac{{\lambda }_{0}}{hc}$ denotes the reciprocal of laser photon energy (photons per J). *N*_*k*_ denotes the fluorophore concentration (g cm^−3^). σ_*k*_ denotes the cross-section of each fluorophore (cm^−2^ sr^−1^ mg^−1^). Δ*R* denotes the optical path in sample (cm)*. ξ* denotes the geometric overlap factor*. η*(*λ*) denotes the total receiver efficiency at *λ*_em_ *=* *λ*. ${\alpha }_{{\lambda }_{0}}$ and *α*_*λ*_ denote the extinction and emission coefficients at *λ*_ex_ = *λ*_0_ and *λ*_em_ = *λ*, respectively^10^. Hence, the fluorescence decay obeys Beer’s law. With the known linear property of fluorescence emission, the trilinear property of TRES is proved and therefore could be decomposed by PARAFAC.

### PARAFAC

PARAFAC is a decomposition method for multi-way data, whose function conceptually can be compared to bilinear principal component analysis (PCA)^20^. PARAFAC generalizes PCA to higher order arrays, and one obvious advantage of the PARAFAC method is the uniqueness of the solution^20^. The rotation problem occurring in bilinear models can be overcome if the appropriate number of factors is selected^20,21^.

PARAFAC decomposed the TRES array into a series of trilinear factors, m, consisting of one score vector and two loading vectors^20^. The principle of PARAFAC is minimization of the sum of squares of the residual *e*_*ijk*_, Eqn. (2) can be given as follows:

2 $${x}_{ijk}={\sum }_{m=1}^{M}{a}_{im}{b}_{jm}{c}_{km}+{e}_{ijk}$$

where *x*_*ijk*_ refers to the fluorescence intensity of sample i, j refers to emission wavelength, k refers to decay time. Next, the TRES array was decomposed, with each factor m comprised of sample scores *a*_*im*_, emission loadings *b*_*jm*_, and decay time loadings *c*_*km*_. The residual *e*_*ijk*_ containing the variation was not incorporated into the PARAFAC model.

In PARAFAC, the core consistency diagnostic was conducted first to select the appropriate number of factors. Next, split-half analysis was performed for validation^20^. The concept of split-half analysis is to divide the data set into two halves and then perform PARAFAC on each of the halves separately. Because of the unique solution of the PARAFAC, almost the same results will be obtained from both halves compared with the non-split mode, e.g., emission and decay mode on both data sets. In this study, PARAFAC was performed to obtain the sample scores as the input of the calibration model.

## Materials and Methods

### Samples

PO, SO and CO were purchased from local markets or online (Table S1, Supplementary data). To guarantee both the quality and the botanical origin, only the brands with good reputation were considered. Note that refining process may result in some unpredictable influence, and only extra virgin grade oils were selected. The adulterated samples were prepared by adding PO or SO into CO at amounts in the range of 0–50% (v/v) with a gradient of 1%. Next, fifty one adulterated samples were generated for each adulterant. These two series of adulterants were called PCO and SCO for short in this study. To avoid inner filter effects, samples were dissolved by mixing 50 mg of the oil into 9.95 mL of n-hexane. Thus, the optical density of samples at the excitation wavelength did not exceed 0.1^22^. All samples were stored under −4 °C in a refrigerator prior to analysis.

### Fluorescence measurements

TRES measurements in the visible wavelength region were performed using HORIBA Scientific DeltaFlex3 (HORIBA, Inc., JPN) equipped with a TBX-07C detector and a 370-nm spectral LED excitation source under 20 °C. TRES was obtained by uniformly increasing the wavelength and measuring the fluorescence decay for equal integration time via the Data-station control and acquisition software. The spectral region ranged from 380 to 600 nm with 5 nm interval. Each sample was contained in the 10-mm fused-quartz cuvette. Equivalent emission spectra were obtained by combining all the temporal data of TRES. Fluorescence decays of PO, SO and CO employing a 370-nm excitation wavelength and a 440-nm cut-off filter were measured. Reconvolutions of these fluorescence decays were performed for two, three, and four components. Here, these reconvolutions are used to supply advanced information rather than to rigorously attribute spectra and lifetimes to species.

### Data analysis

#### Spectral pretreatment

Prior to statistical analysis, the standard normal variate (SNV) algorithm was adopted to rule out the possibility of baseline shift and global intensity change. The Savitzky-Golay algorithm with a window of five points was performed in an effort to decrease noise as much as possible. All pretreatments were performed within the emission wavelength’s mode.

#### Decomposition of TRES

TRES spectra of PCO and SCO were gathered first to build the data in the form of a three-way **Z** array (Fig. S2, Supplementary data). The number of PCO and SCO were both 51. The spectral region ranged from 380 to 600 nm with 5 nm interval and the time region was set from 55 to 60 ns with 0.05 ns interval. Hence, the dimension of **Z** was 51 × 44 × 100 for both PCO and SCO. Then, PARAFAC were performed on **Z** of PCO and SCO, respectively.

#### Model calibration

ANN was used to build the model for adulteration analysis. Cross validation and external validation were applied to evaluate the predictive ability of the model. Fifty-one adulterated samples were arranged by the adulteration concentration and then the training set (n = 51) was composed. Another eleven adulterated samples at amounts in the range of 0–50% (v/v) with a gradient of 5%, were prepared and added to the test set (n = 11). The values of root mean square error of calibration (*RMSEC*), root mean square error of cross-validation (*RMSECV*), root mean square error of prediction (*RMSEP*) and the corresponding coefficients of determination for calibration (${R}_{c}^{2}$), cross validation (${R}_{cv}^{2}$) and prediction (${R}_{p}^{2}$) were calculated.

#### Statistical analysis

To determine the LOD, Duncan’s multiple test was applied to determine the significance of the differences between the intensities of TRES spectra obtained for the genuine and adulterated samples. If the intensities of TRES spectra for the genuine and adulterated samples (lowest in adulteration concentration) differed significantly (*p* < 0.05, according to the Duncan’s multiple test), then we obtain the LOD^23^.

### Ethical Approval

This article does not contain any studies with human participants or animals performed by any of the authors.

## Electronic supplementary material

Supplementary Information

## Data Availability

The datasets generated during and/or analyzed during the current study are available from the corresponding author on reasonable request.
