# Supplementary material for: A novel method for detection of camellia oil adulteration based on time-resolved emission fluorescence
Source: Sci Rep. 2018 Sep 13;8:13784. doi: 10.1038/s41598-018-32223-6 (PMC6137195; doi:10.1038/s41598-018-32223-6)
Supplement: Supplementary file 1 — Supplementary Information [file 41598_2018_32223_MOESM1_ESM.pdf]

Supplementary Data

A novel method for detection of camellia oil adulteration based on time-resolved emission fluorescence

Hui Chen [1]**, Bin Chen** [1,*]**, Daoli Lu** [1]

[1] School of Food and Biological Engineering, Jiangsu University, Zhenjiang, 212013, China

Table S1. Edible vegetable oils investigated in this study.

Classification	Brand	Geographical origin	Source
Peanut oil	Luhua	China	Online retailer
	Jinlongyu	China	Online retailer
	Fulinmen	China	Online retailer
Sunflower oil	Duoli	China	Local supermarket
	Changshouhua	China	Local supermarket
	Kuiwang	China	Local supermarket
Camellia oil	Jinhaoyuan	China	Local supermarket
	Shanrun	China	Online retailer
	Honghao	China	Local supermarket

Table S2. ANN statistics for cross and external validation of concentration of adulterant analysis. PCO: Camellia oil adulterated with peanut oil. SCO: Camellia oil adulterated with sunflower oil. *RMSEC*: root mean square error of calibration. *RMSECV*: root mean square error of cross validation. *RMSEP*: root mean square error of prediction. R_c^2, R_{cv}^2 and R_p^2 stand for the corresponding coefficients of determination for calibration, cross validation and prediction, respectively.

Sample	Calibration		Full cross-validation		External validation	
	RMSEC	R_c^2	*RMSECV*	R_{cv}^2	*RMSEP*	R_p^2
PCO	1.1	0.991	1.8	0.981	2.4	0.968
SCO	1.2	0.994	1.5	0.983	1.7	0.971

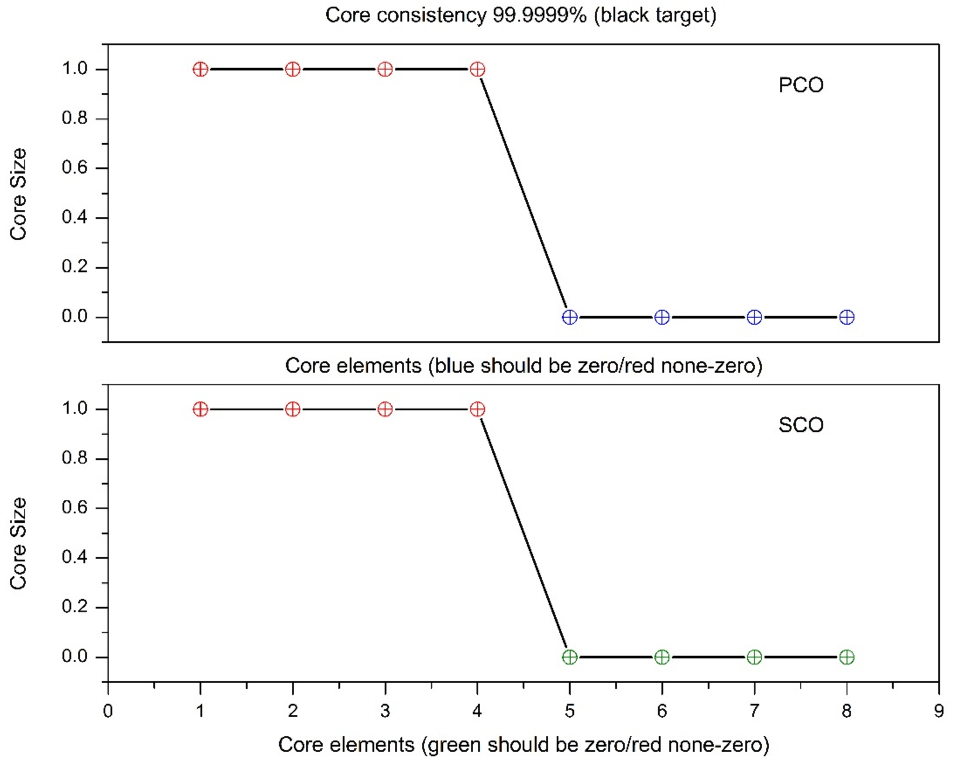

Figure S1. The results of core consistency obtained in the PARAFAC decomposition of TRES arrays of PCO (up panel) and SCO (bottom panel), respectively. The appropriate number of factor m is 4 for both PCO and SCO, agreeing with the number of red point shown in the plots.

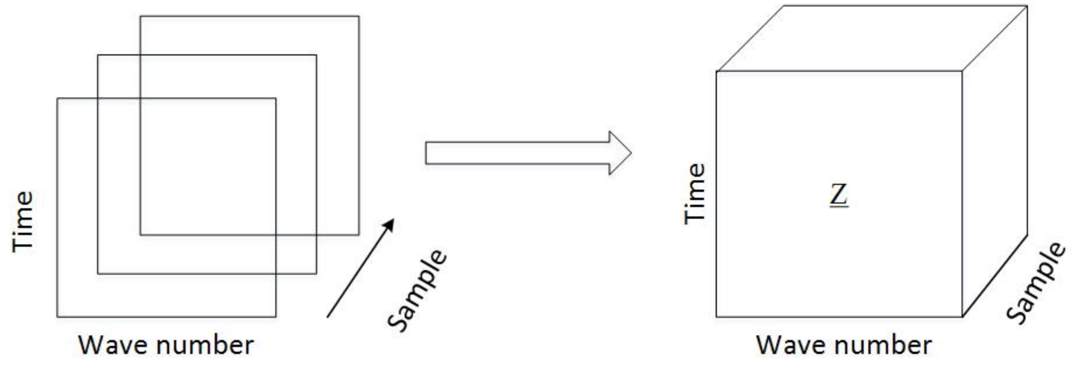

Figure S2. Three-way **Z** array data, for both PCO and SCO.

Equation S1. The ANN model for adulteration analysis. The words "purelin" and "tansig" are all function instructions used in the Matlab. Capital "W" refers to the weighting coefficient and letter "b" refers to the threshold value.

$$\text{Output}= \text{purelin}\ (W_{23} \times \text{tansig}(W_{12} \times \text{Input} + b_2) + b_3).$$

For PCO

W_{12}= [50.9127 -15.1614; 56.4326 -71.4642; 1.4503 -0.1406],

b_2= [-22.4266; -58.8533; 0.1091],

W_{23}= [0.2058 -0.1973 0.6880],

b_3= -0.3500;

For SCO

W_{12}= [33.752 -18.5431; 87.2341 -23.5871; 5.8356 -0.0016],

b_2= [-13.1754; -43.2171; 0.6631],

W_{23}= [0.1314 0.5187 -0.4375],

b_3= 0.5755.
